# A high-efficiency palmprint recognition model integrating ROI and Gabor filtering

**DOI:** 10.1371/journal.pone.0323373

**Published:** 2025-06-02

**Authors:** Nan Zhang, Maolong Xi

**Affiliations:** School of Control Engineering, Wuxi Institute of Technology, Wuxi, China; Islamia University of Bahawalpur: The Islamia University of Bahawalpur Pakistan, PAKISTAN

## Abstract

Palmprint recognition, as a biometric recognition technology, has unique individual recognition and high accuracy, and is broadly utilized in fields such as identity verification and security monitoring. Therefore, a palm print recognition model that integrates regions of interest and Gabor filters has been proposed to solve the problem of difficulty in feature extraction caused by factors such as noise, lighting changes, and acquisition angles that often affect palm print images during the acquisition process. This model extracts standardized feature regions of palmprint images through the region of interest method, enhances texture features through multi-scale Gabor filters, and finally uses support vector machines for classification. The experiment findings denote that the region of interest model performs better than other methods in terms of signal-to-noise ratio and root mean square error, with a signal-to-noise ratio of 0.89 on the GPDS dataset and 0.97 on the CASIA dataset. The proposed model performs the best in recognition accuracy and error convergence speed, with a final accuracy of 95%. The proposed model has the shortest running time, less than 0.4 seconds in all groups, especially less than 0.3 seconds in Group 4, demonstrating high recognition efficiency. The research conclusion shows that the palmprint recognition method combining regions of interest and Gabor filters has high efficiency and performance, and can effectively improve recognition accuracy.

## 1. Introduction

Palmprint recognition, as a biometric recognition technology, has received widespread attention in the last few years due to its high recognition accuracy and unique biometric features. Compared with traditional biometric technologies such as fingerprints and irises, palmprints have individual uniqueness in terms of palm size, line shape, and pattern distribution, thus showing great potential for applications in identity verification, security monitoring, financial payments, and other fields [1,2]. The basic principle of palmprint recognition technology is to extract the geometric and texture features of the palm, establish a unique identity model, and achieve accurate identification of individual identity. However, in the process of obtaining palmprint images, the images are often affected by lighting, angle changes, and noise interference, resulting in a large amount of unnecessary background information and noise data, which affects the accuracy of palmprint feature extraction and recognition. At present, palmprint recognition technology is mostly based on feature extraction methods, including automatic feature extraction grounded on deep learning and traditional image processing methods [3,4]. Deep learning methods typically require large amounts of training data and computational resources, although they have achieved significant results. Traditional methods focus on optimizing recognition performance through image preprocessing and feature engineering, but still face issues of noise interference and information loss [5]. Therefore, to address the issues of noise interference and information loss, a palmprint recognition model that integrates Region of Interest (ROI) and Gabor filters has been proposed. Normalized feature regions of palmprint images are extracted by ROI method, texture features are enhanced by multiscale Gabor filters, and finally classification is performed using Support Vector Machine (SVM). The innovation of the research lies in proposing a palmprint recognition model that combines ROI standardization extraction and Gabor filter enhanced features, which can improve recognition accuracy on the basis of denoising and enhancing texture features. In addition, SVM is utilized for feature classification further optimizes recognition efficiency and classification accuracy. The research aims to maintain high recognition performance under different lighting, angle, and noise conditions using this method, providing a reliable solution for practical applications.

Compared to existing methods, many studies focus on static weight setting or achieving modality fusion through simple algorithms, while ignoring the mutual influence between modalities and the differences in image content. The biggest difference in the research is the introduction of a fusion framework based on Gabor filters, which not only considers the low-level features of images, but also learns the fusion method of high-level semantic information through deep learning models, thereby further improving the accuracy and robustness of multimodal image fusion. The research content is mainly divided into four sections. The first section is a review of other scholars’ research topics on palmprint recognition. The second section is a brief description of the algorithms mainly used in this study, and the third section is the model results obtained by using algorithms for research, and the analysis of the results. The fourth section is a summary of all the above studies and prospects for future research.

## 2. Related works

Gabor filter is a linear filter applied for image processing and signal analysis, widely applied in fields such as image recognition, texture analysis, feature extraction, etc. Wang L. et al. proposed a refined mean shift image segmentation method, founded upon Gabor texture features, to enhance the accuracy of high-resolution remote sensing image segmentation. The research results indicated that this method extracted features through multi-scale and multi-directional Gabor filters and performed well on two remote sensing datasets, corresponding to higher global segmentation quality index and lower error rate, confirming its potential in remote sensing image segmentation [6]. Das P et al. proposed a method based on grey wolf optimization Gabor filtering, spatial fuzzy c-means clustering, and non subsampling shear wavelet transform to improve the computerized evaluation of brain tumor recognition and identification processes, combined with hierarchical correlation and topological structure feature analysis. The research results indicated that the model exhibited high accuracy on multiple datasets, which can help improve research, predict growth rates, develop treatment plans, and monitor clinical trials [7]. Saeed M K et al. designed a disease diagnosis model grounded on deep learning to accelerate the analysis of lung and colon cancer and improve diagnostic accuracy. This method used Gabor filtering to preprocess images, generated feature vectors using Faster SqueezeNet, and classified using convolutional neural networks. The research results indicated that the designed model performed well on medical image datasets, with an accuracy of up to 99.54% [8]. Abdel-Moneim M et al. designed a deep learning classification technique that combines Gabor filtering and thresholding to improve the accuracy of automatic modulation classification, implemented using convolutional filters. The research results showed that the automatic modulation classification system achieved significantly improved classification accuracy for seven modulation types within the signal-to-noise ratio (SNR) range of -10 to 30dB, and achieved a significant classification accuracy of about 100% at an SNR of 10dB. It was suitable for adaptive modulators in applications such as the Internet of Things [9].

Xu D M et al. designed a coupled prediction model to raise the accuracy of runoff prediction. The model utilized a decomposition approach that involved the improved full-integrated empirical model decomposition combined with wavelet decomposition, and employed an SVM that had been optimized by the seagull optimization algorithm for prediction. The research results demonstrated that the model exhibited the optimal performance in terms of monthly runoff forecasting for the Hongjiadu and Manwan reservoirs, characterized by the highest prediction accuracy and the lowest prediction error [10]. Ding W et al. designed a multi-objective optimization method based on a swarm intelligence algorithm using an SVM and a multi-objective particle swarm optimization algorithm to enhance the quality of injection moulding for thin-walled plastic components, thereby addressing issues related to warping, deformation and volume shrinkage. The research results demonstrated that following the optimization process, warpage deformation was reduced by 40.9%, the volume shrinkage rate was reduced by 18.1%, and the injection molding quality was significantly improved [11]. Neethu P S et al. put forward a novel approach for the classification of gestures, utilizing SVM classification to enhance the precision of gesture categorization. The distance transformation method was applied to detect the centre point of the segmented palm area. The findings of the study demonstrated that this method yielded high levels of sensitivity, specificity, and accuracy across multiple datasets, making it well-suited for real-time gesture applications in environments with multiple backgrounds [12]. Yang Z et al. proposed a physically driven spectrum consistent federated learning method, PSF Palm, to alleviate the challenges and privacy protection issues of existing palmprint verification methods in cross spectrum verification. The research results indicated that PSF Palm ensured spectrum consistency and protected data privacy through grouping, anchor model introduction, and spectrum consistency loss design, and demonstrated convincing performance even in limited training data [13]. To fully utilize the high-order texture information of palmprints, Yang Z et al. used second-order textures for palmprint recognition for the first time. They extracted second-order textures through convolution operations and generated second-order texture co-occurrence codes. The research results indicated that the proposed method performed well on various databases. Furthermore, the proposed multi-level texture co-occurrence code could more comprehensively describe palmprint features and had significant accuracy performance compared to state-of-the-art methods on all public databases [14].

In summary, Gabor filters have been broadly utilized in image processing and signal analysis, especially in the texture analysis, image recognition, and feature extraction, where significant achievements have been made. Gabor filters play an important role in human pose estimation and texture classification due to their excellent localization properties and advantages in multi-scale and multi-directional processing. However, in practical applications, especially when dealing with complex images and other high difficulty tasks, there are still some challenges, such as computational efficiency and model optimization issues. Therefore, the study targets to raise the accuracy of image feature extraction and further optimize the palmprint recognition model by combining Gabor filters with deep learning networks, promoting the in-depth development of image processing technology in a wider range of application fields.

## 3. Methods

The first section provides an overview of the standardization process for extracting ROI from palmprint images using the ROI method, including palm foreground segmentation, image smoothing, white balance processing, edge detection, and ROI standardization extraction. The second section proposes a palmprint recognition model based on Gabor filters, which achieves effective recognition of palmprint images through biomimetic texture enhancement, feature detection and encoding, and SVM classification. In summary, the method proposed in this study consists of an ROI extraction module and a Gabor-based recognition model.

### 3.1. Standardized ROI extraction method for palmprint images

**Fig 1:**
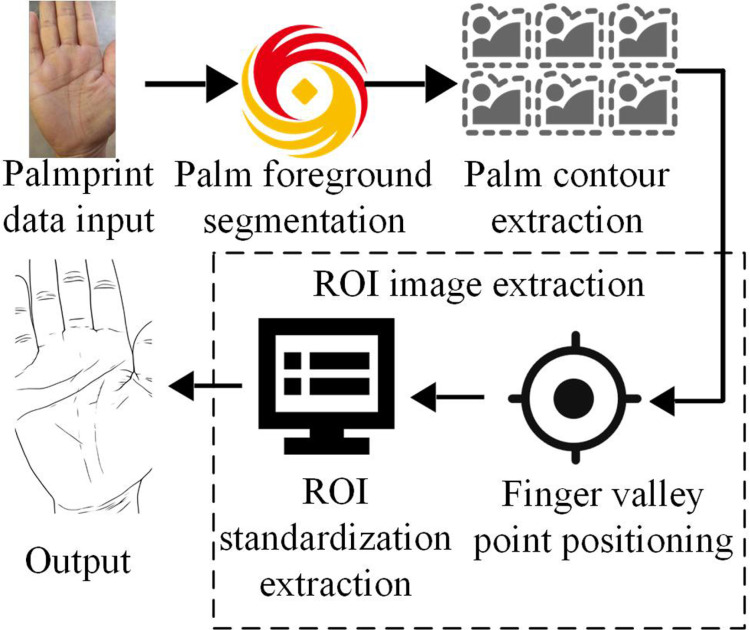
SPEM-ROI-based palmprint extraction process.

Palmprint recognition, as a biometric recognition technology, has received widespread attention in recent years. Compared with traditional biometric technologies such as fingerprint recognition and iris recognition, palmprint recognition has higher recognition accuracy and unique biometric features, especially in terms of individual uniqueness in palm size, line shape, and pattern distribution. This makes palmprint recognition have great potential for applications in fields such as identity verification, security monitoring, and financial payments. The acquisition of palmprint images requires the use of information from the palm area, but the palmprint images obtained through this method will contain a large amount of noisy data, which will affect the main features of the palmprint. Therefore, it needs to process the palmprint images to extract the ROI with the most feature images. For the extraction of palmprint ROI, the method is named SPEM-ROI, and its steps are shown in Fig 1.

In Fig 1, the palmprint image is first subjected to palm foreground segmentation, then the input image data is applied to extract the palm contour, and finally the ROI image is extracted. ROI image extraction contains two steps: first, locate the points between the fingers, then perform ROI standardization extraction, and finally input the extracted image [15,16]. In palm foreground segmentation of palmprint images, the presence of image noise can make the palm contour unclear. Therefore, before palm foreground segmentation, smoothing filtering is first performed, and its expression is shown in Equation 1.


$$O(x,y)=\frac{1}{k^{2}}\sum_{i=-\frac{k}{2}}^{\frac{k}{2}}\sum_{j=-\frac{k}{2}}^{\frac{k}{2}}I(x+i,y+i)$$ (1)

**Fig 2:**
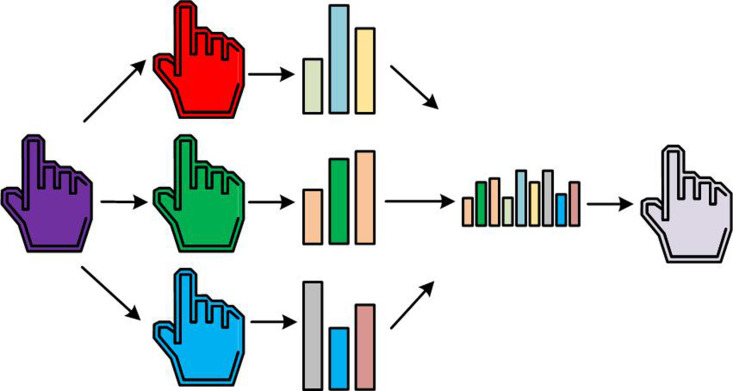
Schematic diagram of automatic WB algorithm.

In Equation 1, O represents the smoothed filtered value. (x,y) refers to the position of the current pixel, I(x+i,y+i) is weighted averaged for each pixel within the window, and k denotes the size of the filter. The study adopts non-contact palmprint image for palm foreground segmentation, which extracts the palm area from the captured palmprint image through image processing techniques without touching the palm, and separates it from the background. By using an automatic WB algorithm based on histograms to process images, the purpose of WB is to adjust the color temperature of the image, so that the white areas in the image appear as real white, thereby making the image colors look natural [17]. The core idea of this algorithm is to process the chromaticity space map, calculate the histogram overlap area of each channel, and ultimately obtain the optimal WB state. The process of this method is denoted in Fig 2.

In Fig 2, the chromaticity space map is first obtained, and WB is usually based on the chromaticity space. Firstly, it converts the original image from RGB space to chromaticity space. Each pixel in the chromaticity space map can be decomposed into chromaticity information and brightness information. Among them, the chromaticity information in the chromaticity space map is used to adjust the color balance of the image. Its expression is shown in Equation 2.


$$\left\{ \begin{array}{c} r=\frac{R}{R+G+B}\\ g=\frac{G}{R+G+B}\\ b=\frac{B}{R+G+B} \end{array}\right.$$ (2)

In Equation 2, r, g, and b represent the normalized red, green, and blue channel values, respectively. R, G, and B are the original values of the red, green, and blue channels of a pixel in the image, respectively [18,19]. Then, the histograms of each channel are calculated, which is a way to represent the distribution of pixels in an image. By counting the frequency of appearance of each chromaticity value in the image, the overall color distribution of the image can be reflected. The histogram of any chromaticity channel is shown in Equation 3.


$$h^{c}\left(i\right)=\underset{N}{\overset{x=1}{\sum }}\;\underset{M}{\overset{y=1}{\sum }}\;\frac{1}{N\times M}\left\{ \begin{array}{c} 1,c\left(x,y\right)=i\\ 0,Other \end{array}\right.$$ (3)

In Equation 3, $h^{c}\left(i\right)$ represents the histogram of any one of the chromaticity channels r, g, and b. M and N denote the length and width of the image. Next, the overlapping area of the chromaticity histogram is calculated. In WB processing, the goal is to find a chromaticity transformation that makes the image tone distribution approach the state of “neutral white” [20]. To this end, the histogram overlap area between different channels is calculates to determine how to adjust colors. Its expression is shown in Equation 4.


$$H\left(h^{r},h^{g},h^{b}\right)=\underset{i=1}{\overset{n}{\sum }}\;h^{r}(i)⋂h^{g}(i)⋂h^{b}(i)$$ (4)

In Equation 4, h represents the value of the grayscale level in the channel. Finally, the optimal state is found to correct the gains of the R, G, and B color channels, as expressed in Equation 5.


$$\mathrm{\backslash arg}\underset{k_{R},k_{G},k_{B}}{\max}\;H\left(h^{r}\left(k_{R}\right),h^{g}\left(k_{G}\right),h^{b}\left(k_{B}\right)\right)$$ (5)

In Equation 5, k represents the gain parameter. By minimizing the overlap area between chromaticity histograms, color adjustment parameters are optimized to make the color tone distribution of the image closer to the standard white distribution [21,22]. Human skin is concentrated in certain color spaces, so images can be segmented based on the different color components. Firstly, the color component is selected and the quantitative evaluation of bimodal behavior is studied using Peak-to-Base Index, as expressed in Equation 6.


$$PBI=\frac{H_{1}-H_{0}}{H_{2}-H_{0}}$$ (6)

In Equation 6, $H_{1}$ represents the height of the first peak of the bimodal pattern, $H_{2}$ means the height of the second peak, and $H_{0}$ means the lowest value between the two peaks. Then, the skin pixels are modeled using color components, and finally palm foreground segmentation is performed based on color components. After completing image segmentation, the hand contour is extracted through boundary tracking method. The Canny edge detection algorithm is used for hand contour extraction, and its calculation formula is shown in Equation 7.


$$E\left(x,y\right)=\sqrt{{\left(\frac{\partial I\left(x,y\right)}{\partial x}\right)}^{2}+{\left(\frac{\partial I\left(x,y\right)}{\partial y}\right)}^{2}}$$ (7)

**Fig 3:**
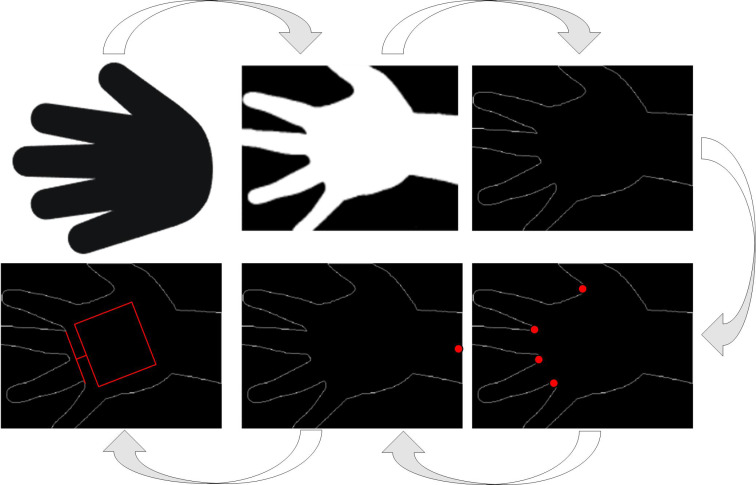
ROI image extraction process.

In Equation 7, E(x,y) means the edge intensity of the image at position (x,y), I means the brightness value, $\frac{\partial I\left(x,y\right)}{\partial x}$ and $\frac{\partial I\left(x,y\right)}{\partial y}$ represent the gradient of image brightness in different directions, respectively. Finally, ROI image extraction is performed, as denoted in Fig 3.

From Fig 3, ROI image extraction mainly consists of two steps, namely the localization of inter finger points and ROI standardization extraction. Firstly, the positioning of each point between fingers is achieved through image segmentation and edge detection techniques to identify the fingers and interfinger regions in the hand image. By utilizing contour extraction and keypoint detection algorithms, accurate identification of finger roots, fingertips, and interphalangeal points is achieved, which provide a foundation for subsequent ROI extraction [23,24]. By connecting adjacent finger points, the spatial distribution between fingers is determined, providing a geometric basis for delineating the palm and finger areas. Next, ROI standardization extraction is performed by determining a rectangular box based on the position of the interphalangeal points, enclosing the ROI, and standardizing the extracted area to a uniform size through scaling operations. This standardization process ensures that images of different resolutions can be uniformly input into subsequent analysis systems. After standardizing ROI images, preprocessing operations such as denoising and lighting compensation are usually required to improve image quality and feature extraction accuracy. Finally, the processed ROI image can be input into models such as palmprint recognition and gesture recognition for further analysis.

### 3.2. Palmprint recognition model based on Gabor filter

**Fig 4:**
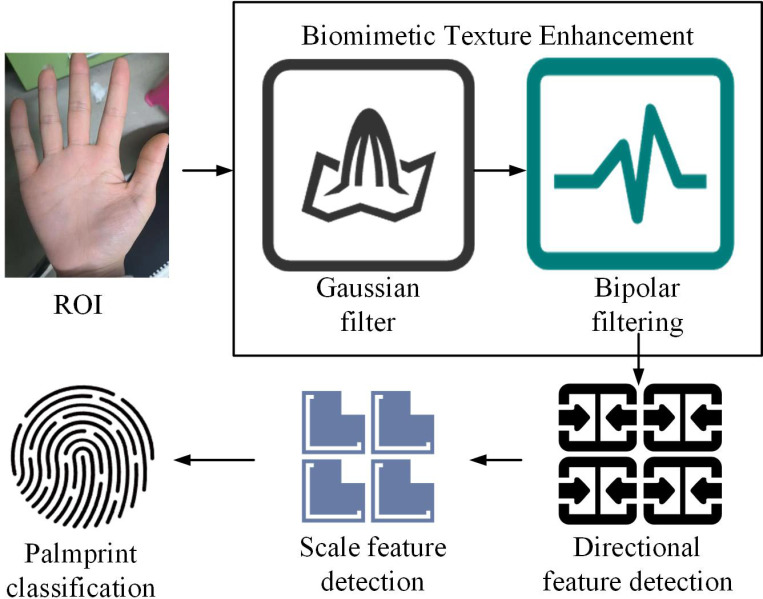
Structure of palmprint recognition model based on Gabor filter.

In view of the fact that existing palmprint features are all convolved from palmprint ROIs, and the method proposed in the first section extracts palmprint ROIs that contain a lot of noise, this study uses Gabor filters combined with biomimetic texture enhancement to recognize palmprints. The structure is shown in Fig 4.

In Fig 4, the model is composed of three stages, namely biomimetic texture enhancement, feature detection and encoding, and palmprint image classification. Firstly, Gaussian filtering is applied to the input palmprint ROI image using sliding window convolution, smoothing the image and reducing noise to ensure clarity for subsequent feature extraction [25,26]. Next, a bipolar filter is synthesized using vertical and horizontal sub filters to simulate the response of bipolar cells in the human visual cortex to lines. Processing the smoothed image through a bipolar filter can enhance the texture features in different directions of the palmprint image, improve the expression of local features in the image, and thus enhance the robustness and discriminability of feature extraction. Subsequently, the direction and scale features of the image are extracted using a multi-scale Gabor filter bank. Gabor filters can effectively capture the detailed information of palmprint images, and filters of different scales provide multi-level texture features for the images, making the extracted features more robust [27]. These feature codes are combined to form the feature codes of the palmprint image, representing the core information of the image. Finally, by calculating the angular distance to measure the similarity between any two palmprint images, the classification of palmprint images is completed. The angular distance quantifies the differences between images, helping the classifier effectively distinguish different palmprint samples and achieve palmprint recognition. Gaussian filtering is applied to process the ROI map. Its expression is shown in Equation 8.


$$G_{s}(x,y,\sigma )=\frac{1}{2\pi \sigma^{2}}\exp(-\frac{x^{2}+y^{2}}{2\sigma^{2}})$$ (8)

In Equation 8, $G_{s}(x,y,\sigma )$ refers to the image output after Gaussian filtering. σ represents the standard deviation of Gaussian distribution, which controls the smoothness of the filter. x and y are the spatial coordinates of the filter, representing the offset between the filter and the corresponding position of the image. Bipolar filtering is an image processing method commonly used to enhance edge or texture features in images [28]. Its working principle is similar to simulating bipolar cells in the human visual cortex, which have contrast sensitive characteristics and can respond to changes in light intensity, especially at the edges of images. From the perspective of visual mechanism, bipolar cells are located in the retina, connecting photoreceptor cells and ganglion cells, mainly responsible for transmitting photoreceptor cell signals to the brain and performing preliminary image processing in this process. Bipolar cells enhance the contrast of images to highlight the light and dark boundaries, thereby helping the brain better recognize the shape and contour of objects. It can sensitively reflect changes in light intensity in the image, especially in the edge areas of objects, by enhancing these changes to highlight the edge features of the image [29,30]. Bipolar cells respond to changes in local light intensity, enhancing detailed information in the image, such as contours of lines and shapes. By processing the changes in light intensity at different brightness levels, bipolar cells enhance the contrast in images, helping the visual system perceive the edges of objects. Inspired by the above mechanism, the study adopts bipolar filters to highlight edge responses in images. Bipolar filters use contrast operations to highlight details in images, especially for processing texture information such as lines and edges [31,32].

A bipolar filter uses two sub filters. One is a vertical sub filter and the other is a horizontal sub filter. The combination of the two forms the final bipolar filter, whose expression is shown in Equation 9.


$$B(x,y)=\frac{G_{v}(x,y)-G_{h}(x,y)}{G_{v}(x,y)+G_{h}(x,y)}$$ (9)

In Equation 9, B(x,y) is the image processed by a bipolar filter, $G_{v}(x,y)$ denotes the vertical filter response, and $G_{h}(x,y)$ denotes the horizontal filter response. The expressions for vertical and horizontal filters are shown in Equation 10.


$$\begin{array}{c} \left\{ \begin{array}{cc} @\;\mathrm{-10pt}ll & G_{v}(x,y)=\underset{i=-1}{\overset{1}{\sum }}\;\underset{j=-1}{\overset{1}{\sum }}\;I(x+i,y+j)\cdot V(i,j)\\ & G_{h}(x,y)=\underset{i=-1}{\overset{1}{\sum }}\;\underset{j=-1}{\overset{1}{\sum }}\;I(x+i,y+j)\cdot H(i,j) \end{array}\right. \end{array}$$ (10)

**Fig 5:**
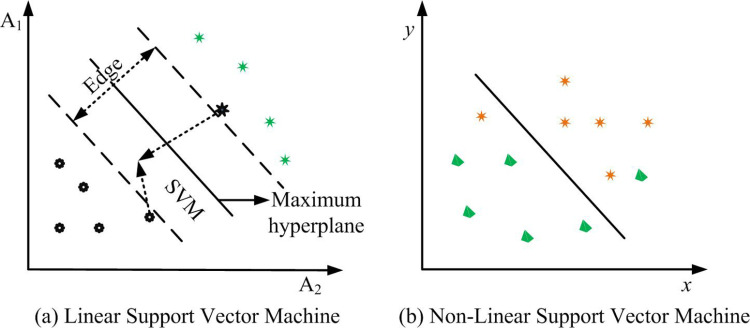
SVM schematic diagram.

In Equation 10, I(x,y) represents the input image, V(i,j) means the vertical convolution kernel, and H(i,j) means the horizontal convolution kernel. By calculating the angular distance to measure the similarity between any two palmprint images, the classification of palmprint images is completed. The angular distance quantifies the differences between images, helping the classifier effectively distinguish different palmprint samples and achieve contact-based palmprint recognition. However, this method requires a large amount of computation and high equipment requirements, so SVM is chosen for palmprint feature recognition in the study. SVM mainly includes linear SVM and nonlinear separable SVM, as shown in Fig 5.

**Fig 6:**
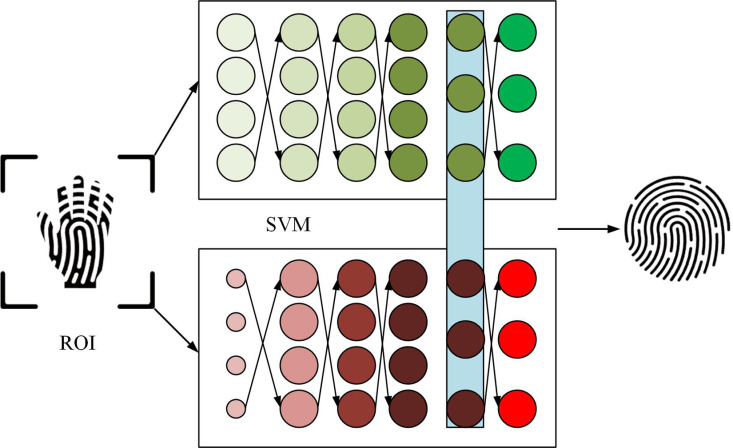
Gabor-SVM model structure.

In Fig 5, in SVM, hyperplanes are used to separate decision boundaries of different categories. In 2D space, a hyperplane is a straight line; In 3D space, a hyperplane is a plane; In higher dimensions, a hyperplane is a hyperplane. The support vector is the sample point closest to the decision boundary. These points take an important part in defining the hyperplane, hence they are called support vectors. Interval refers to the distance to the nearest support vector. SVM finds the optimal separation hyperplane by maximizing this interval. Linear separable SVM effectively classifies different samples by searching for the largest edge hyperplane in the feature library space. The study uses linear SVM as the final classifier. The final model structure is denoted in Fig 6.

In Fig 6, firstly, Gaussian filtering is applied to the palmprint ROI image using sliding window convolution. Then, a bipolar filter is synthesized using vertical and horizontal sub-filters to simulate the response of bipolar cells in the human visual cortex. The image is processed using the bipolar filter to highlight texture features in different directions, enhance the local expression of the image, and improve the robustness and discrimination of feature extraction. Subsequently, multi-scale Gabor filter banks are utilized to extract the directional and scale features of the image. Gabor filters can effectively capture the details of palmprint images, and filters of different scales provide multi-level texture information, enhancing the stability of features. These features are encoded and combined to form the feature code of the palmprint image, representing the core information of the image. Finally, SVM is used to train and classify the feature codes. SVM can effectively distinguish different palmprint images and complete the classification task of palmprints by maximizing the inter class interval, achieving high-precision contact-based palmprint recognition.

By extracting standardized feature regions through ROI, background interference is removed, while Gabor filters enhance texture features and improve recognition accuracy. In addition, using support vector machine (SVM) for classification effectively improves recognition efficiency and accuracy, especially under conditions of noise and lighting changes, solving the image quality problems caused by lighting, angle changes, and noise interference in palmprint recognition.

## 4. Results

### 4.1. Analysis of the effect of standardized roi extraction method for palmprint images

**Fig 7:**
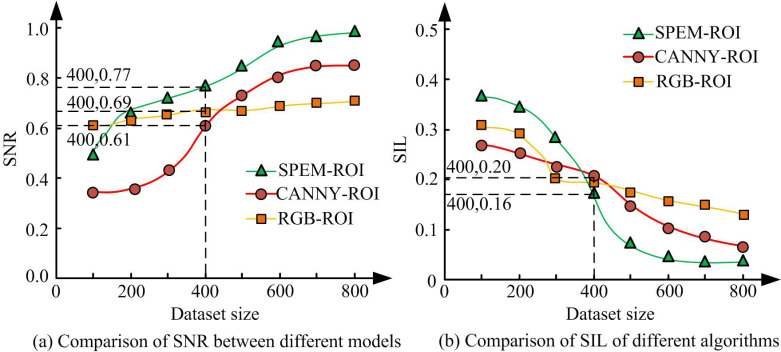
Comparison of SNR and SIL among various models.

The hardware configuration used in the study was Intel Core i7-13900KF CPU, NVIDIA Geforce RTX4090D GPU, 16GB of VRAM, and 64GB of RAM. The dataset adopted Grupo de Procesado de Señales (GPDS) palmprint public database and CASIA palmprint public database. GPDS contained palmprint images from 350 different users. Each user had 4 different palm images, collected under different conditions with a resolution of 320 × 240. The images in the dataset were collected under different conditions, including different lighting, angles, etc. The CASIA palmprint public dataset contained approximately 4000 palmprint images from over 100 different individuals. Each individual contained multiple palm images and multiple subsets, including single-palm images, multi-angle images, images under different lighting conditions, etc. The study used RGB-ROI and CANNY-ROI as comparison models to compare with the raised model, and the findings are denoted in Fig 7.

**Fig 8:**
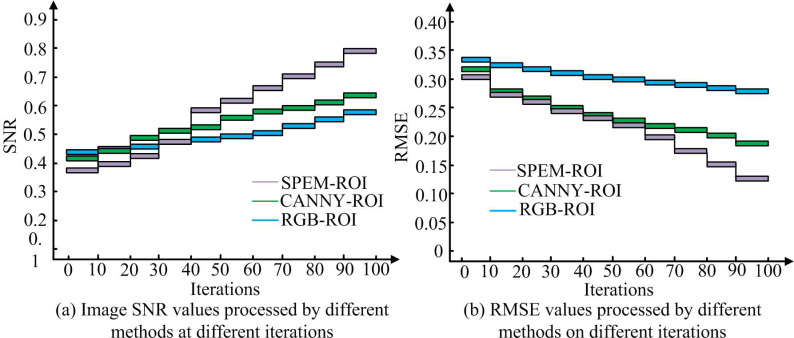
Performance analysis of various models at different iterations.

Fig 7a and b show a comparison of the SNR and structural information loss (SIL) of images processed by various algorithms. In Fig 7a, with the increase of dataset size, the SNR of the SPEM-ROI model continued to rise, demonstrating high robustness. The SNR of the CANNY-ROI model gradually stabilized from 0.69 when the dataset size reached 400. The SNR of RGB-ROI model was relatively low and the variation amplitude was small. When the data volume was 400, the SNR value of SPEM-ROI was 0.77, which was higher than the corresponding values of CANNY-ROI and RGB-ROI. From Fig 7b, the SIL of SPEM-ROI continued to decrease as the dataset size increased, gradually decreasing from 0.20 when the dataset size was 400 to 0.16. However, the SIL changes of CANNY-ROI and RGB-ROI were relatively small and always higher than SPEM-ROI. Especially when the data volume was small, the SIL of SPEM-ROI decreased faster, indicating its significant robustness advantage on small-scale datasets. The performance of each model under different iteration times was analyzed, and the findings are indicated in Fig 8.

**Fig 9:**
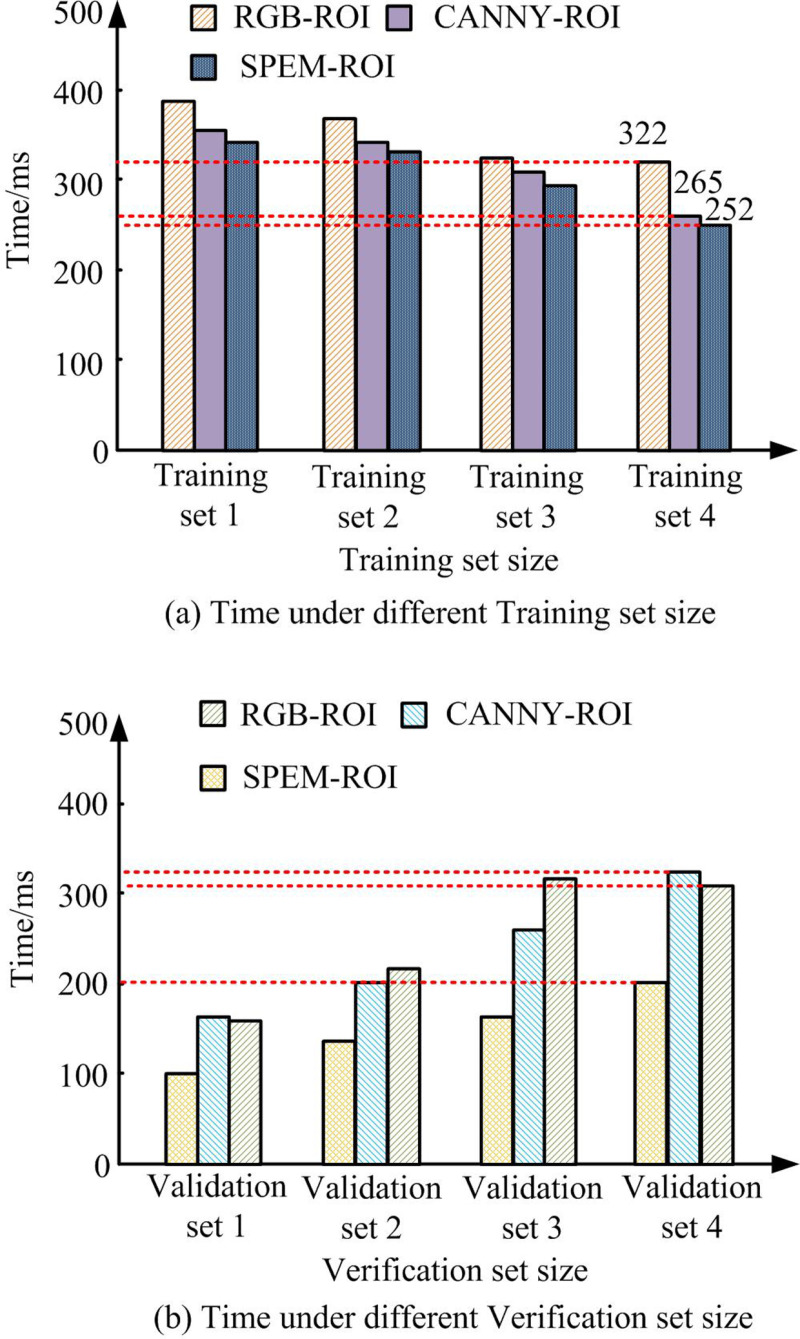
Analysis of processing time for each model.

Fig 8a and b show the changes in SNR and root mean square error (RMSE) of each model at different iterations. In Fig 8a, with the increase of iteration times, the SNR of all three methods significantly improved, but the improvement amplitude and final value of SPEM-ROI were higher. At the 50th iteration, the SNR of SPEM-ROI reached 0.7, significantly better than CANNY-ROI’s 0.6 and RGB-ROI’s 0.55. At the 100th iteration, the SNR of SPEM-ROI further improved to nearly 0.9, while CANNY-ROI and RGB-ROI remained stable at around 0.75 and 0.65, respectively. From Fig 8b, in the early stage of iteration, the RMSE of all methods rapidly decreased, with SPEM-ROI showing the fastest decrease in RMSE, from an initial value of 0.35 to about 0.15. In the later stage of iteration, the decrease in RMSE tended to be gentle, and the RMSE of SPEM-ROI at the 100th iteration was only about 0.13, significantly lower than the 0.18 of CANNY-ROI and 0.27 of RGB-ROI. The processing time of each model was analyzed, and the outcomes are indicated in Fig 9.

Fig 9a and b show the comparison of training time and validation time for each model under different training set sizes. According to Fig 9a, as the size of the training set increased, the training time of all three methods showed an increasing trend, but the RGB-ROI method always had the highest training time. In training set 4, the training time for RGB-ROI was 322 ms, while CANNY-ROI and SPEM-ROI were approximately 264 ms and 252 ms, respectively. SPEM-ROI took the least amount of time across all training set sizes. In Fig 9b, the validation time of RGB-ROI and CANNY-ROI increased rapidly, while the growth rate of SPEM-ROI was relatively flat. In validation set 4, the validation time for RGB-ROI exceeded 450 ms, CANNY-ROI was about 400 ms, and SPEM-ROI was only about 320 ms. The verification time of SPEM-ROI was always the lowest, indicating that it can maintain high efficiency during the verification phase. The findings indicated that the proposed model had excellent efficiency and performance. The comprehensive performance of each model was analyzed, and the outcomes are denoted in Table 1.

**Table 1:** Performance analysis of processing models.

Data set	Model	SNR	SIL	RMSE	Training time/ms	Proof time/ms
GPDS	SPEM-ROI	0.89	0.16	0.13	252	321
	CANNY-ROI	0.75	0.23	0.18	264	412
	RGB-ROI	0.65	0.25	0.27	322	467
CASIA	SPEM-ROI	0.97	0.08	0.05	216	284
	CANNY-ROI	0.83	0.15	0.11	228	364
	RGB-ROI	0.73	0.17	0.19	286	414

According to Table 1, SPEM-ROI performed better than the other two methods on both datasets. The SNR of SPEM-ROI on the GPDS dataset was 0.89, much higher than the 0.75 of CANNY-ROI and 0.65 of RGB-ROI. On the CASIA dataset, the SNR of SPEM-ROI was further improved to 0.97, far exceeding the 0.83 of CANNY-ROI and 0.73 of RGB-ROI. In addition, the RMSE of SPEM-ROI performed the best on both datasets, with 0.13 in the GPDS dataset and 0.05 in the CASIA dataset, both significantly lower than CANNY-ROI and RGB-ROI, indicating that it had the smallest error and more accurate model fitting. From the perspective of training time and validation time, SPEM-ROI had a training time of 252ms and a validation time of 321ms on the GPDS dataset, both of which were better than CANNY-ROI and RGB-ROI. Especially during the validation phase, the validation time of SPEM-ROI was significantly shorter, demonstrating higher efficiency. The experiment findings denoted that the SPEM-ROI model exhibited better performance in SNR, SIL, RMSE, and processing time, and its application effect in palmprint ROI extraction was more prominent.

### 4.2. Performance analysis of palmprint recognition model based on Gabor filter

**Fig 10:**
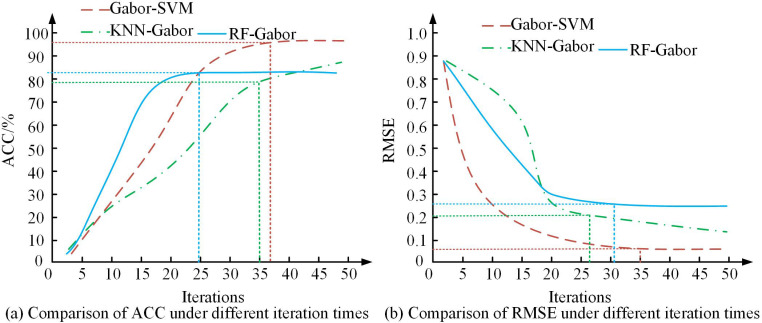
Comparison of ACC and RMSE of various recognition models.

To verify the performance of the Gabor-SVM model, recognition models based on K-Nearest Neighbors (KNN) and Gabor, as well as recognition models based on Random Forest (RF) and Gabor, were introduced and named KNN-Gabor and RF-Gabor, respectively [33]. The KNN algorithm is commonly used in palm print recognition tasks due to its non parametric nature and intuitive similarity measurement mechanism. It calculates the distance between high-dimensional palm print features and achieves identity matching based on nearest neighbor voting, which is particularly suitable for small sample scenarios and 1:1 verification modes. The comprehensive performance of the model was analyzed, and the results are shown in Fig 10.

**Fig 11:**
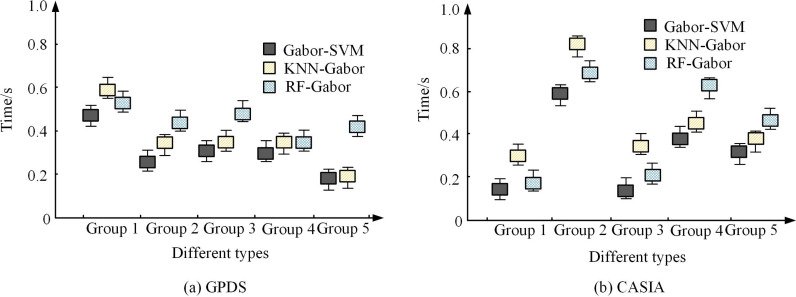
Comparison of processing time for various models.

Fig 10a shows the ACC trend of each model at different iteration times, and Fig 10b shows the RMSE of each model at different iteration times. In Fig 10a, with the increase of iteration times, the ACC of all methods gradually improved. Among them, RF-Gabor had the fastest convergence speed, reaching a stable value after about 25 iterations, and the final ACC was close to 85%. The improvement of KNN-Gabor was relatively slow, and it tended to stabilize after about 35 iterations, with a final ACC of about 85%. The improvement of Gabor-SVM was relatively slow, but its final ACC was relatively high, about 95%. From Fig 10b, the error of Gabor-SVM decreased the fastest, tended to stabilize after about 25 iterations, and the final error was less than 0.1. The error reduction of KNN-Gabor was slightly slower, converging after 35 iterations, with a final error of 0.17. The error convergence of RF-Gabor was earlier, but the final error was relatively high, at 0.27. The experimental results show that the proposed Gabor-SVM performs the best in both accuracy and error convergence speed. The analysis of the recognition time of different models on different types of images in different datasets is shown in Fig 11.

**Fig 12:**
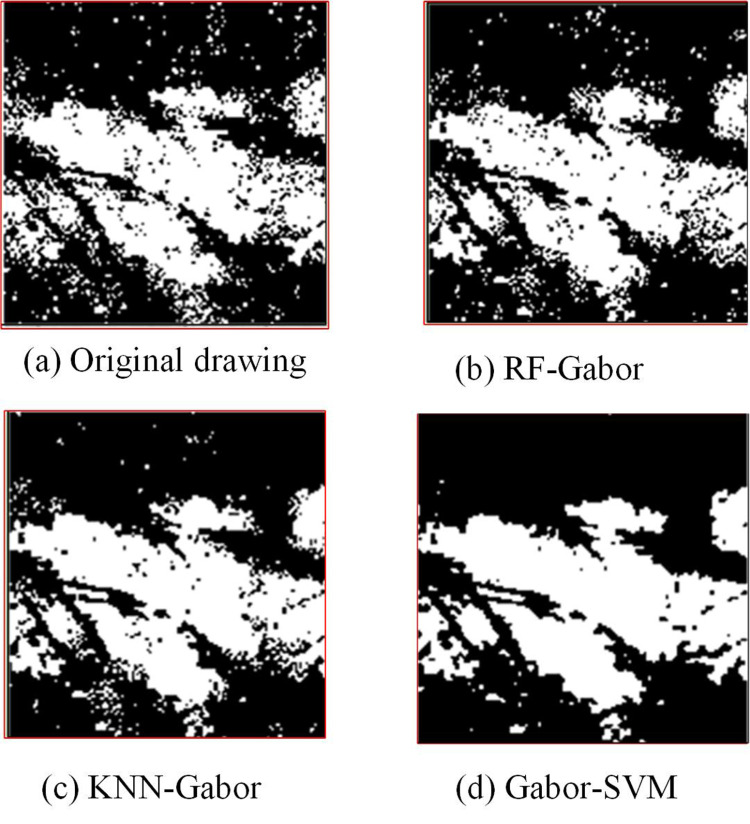
Comparative analysis of palmprints of various models.

Fig 11a and 11b show a comparison of time consumption of different models on different types of palmprint images on the GPDS dataset and CASIA dataset, respectively. According to Fig 11a, Gabor-SVM had the shortest running time, less than 0.4 seconds in all groups, demonstrating high computational efficiency. KNN-Gabor had slightly higher time consumption, with a time range of approximately 0.3 seconds to 0.7 seconds per group. RF-Gabor had the highest running time, especially reaching about 0.7 seconds in Group 1 and Group 3. According to Fig 11b, Gabor-SVM had the lowest time consumption among all groups, especially below 0.3 seconds in Group 4. KNN-Gabor consumed slightly more time per group, ranging from approximately 0.3 seconds to 0.8 seconds. RF-Gabor had the highest time consumption, especially in Group 5, which was close to 0.8 seconds. The experimental results show that the proposed Gabor-SVM model has excellent recognition efficiency. The analysis of actual palmprints is shown in Fig 12.

Fig 12a showcases the original palmprint image, while Fig 12b–12d express the recognition images of RF-Gabor, KNN-Gabor, and Gabor-SVM models, respectively. In Fig 12, the original image was rich in details, but it also contained a large amount of irrelevant information or noise points. After RF-Gabor processing, the texture features of the image were clearer, and many noise points were effectively eliminated while retaining the main texture information. The image processed by KNN-Gabor exhibited a certain balance between preserving texture details and suppressing noise points. Some noise points can still be seen in the image, but the layering and coherence of the texture features have significantly improved. The result after Gabor-SVM processing was compared with other methods. Gabor-SVM’s image processing results were relatively excellent, with details of texture features not lost and noise points completely cleaned up. The experiment findings denoted that the Gabor-SVM model had excellent performance. The comprehensive performance of each model was analyzed, and the outcomes are denoted in Table 2.

**Table 2:** Comprehensive performance analysis of the model.

Data Set	Model	ACC	RMSE	Time	F1 Score	Precision	Recall	AUC	Training Time
GPDS	RF-Gabor	84%	0.31	0.34s	0.81	0.79	0.83	0.87	0.24s
	KNN-Gabor	90%	0.14	0.47s	0.9	0.84	0.91	0.91	0.45s
	Gabor-SVM	94%	0.09	0.69s	0.93	0.91	0.95	0.94	1.23s
	CCNet [34]	92%	0.11	0.72s	0.95	0.84	0.84	0.81	1.11s
CASIA	RF-Gabor	82%	0.33	0.32s	0.79	0.77	0.8	0.84	0.27s
	KNN-Gabor	90%	0.17	0.46s	0.86	0.83	0.84	0.88	0.71s
	Gabor-SVM	92%	0.11	0.71s	0.91	0.88	0.93	0.93	1.27s
	CCNet [34]	89%	0.21	0.98s	0.74	0.79	0.82	0.89	1.12s

According to Table 2, Gabor-SVM had the highest accuracy on the GPDS dataset, reaching 94%, while its RMSE was the lowest, indicating that the difference between predicted results and actual values is minimal. Despite its high computational cost, it had an advantage in classification ACC. KNN-Gabor performed stably with an ACC of 90% and an F1 Score of 0.90. It had a short training time and was suitable for tasks that require a balance between ACC and time. RF-Gabor performed weakly on GPDS with an ACC of 84%, but had the shortest running time and was suitable for scenarios with high real-time requirements. For the CASIA dataset, Gabor-SVM had an ACC of 92% and performed well in precision and error control, despite its longer training time. KNN-Gabor had an ACC of 90% and an F1 Score of 0.86, outperforming RF-Gabor. The ACC of RF-Gabor was 82%, the F1 Score was 0.79, and the calculation time was the shortest.

## Conclusion

Aiming at the issues of noise interference and difficulty in feature extraction in existing palmprint recognition methods, a palmprint recognition model that integrates ROI and Gabor filter was proposed. The standardized ROI of the palmprint image was extracted by SPEM-ROI method, and the texture features were enhanced by multi-scale Gabor filter. Finally, SVM was used for classification. The experiment findings denoted that as the dataset size increased, the SNR of the SPEM-ROI model continued to rise, demonstrating high robustness. The SNR of the CANNY-ROI model gradually stabilized from 0.69 when the dataset size reached 400. The SNR of RGB-ROI model was relatively low and the variation amplitude was small. When the data volume was 400, the SNR value of SPEM-ROI was 0.77, which was higher than the corresponding values of CANNY-ROI and RGB-ROI. The SNR of this model on the GPDS dataset was 0.89, and the RMSE was 0.13. On the CASIA dataset, the SNR was 0.97, and the RMSE was 0.05, both of which were significantly better than the CANNY-ROI and RGB-ROI methods. Meanwhile, the Gabor-SVM model performed excellently in recognition ACC and error convergence speed, with a final ACC of 95%. The research results indicated that the palmprint recognition method combining ROI extraction and Gabor filter had significant advantages in improving ACC, reducing errors, and enhancing efficiency. The contribution of the research lies in proposing a palm pattern recognition model that combines ROI extraction and Gabor filtering, which solves the problems of noise interference and feature extraction difficulties in existing methods. The SPEM-ROI method was used to extract standardized ROIs, enhancing the expressiveness of image features, and multi-scale Gabor filtering was applied to enhance texture features, improving the ACC and robustness of recognition.

### Limitation and future work

There are still certain shortcomings. Firstly, this method requires substantial computational resources. This reliance on deep learning models for feature extraction and fusion necessitates large datasets and significant computing power, which may present challenges for devices with limited resources. Secondly, while deep learning models can adaptively adjust fusion strategies, their performance may still be constrained in low-contrast or high-noise image scenarios, particularly when dataset quality is suboptimal. Additionally, the training process of the proposed method is relatively complex, requiring extensive hyperparameter tuning, thereby increasing the difficulty of algorithm debugging and extending the development cycle. Furthermore, while convolutional neural networks are employed for feature learning, they may not fully adapt to certain image structures, leading to loss of detail or uneven fusion in localized regions. Finally, due to the method’s training on large-scale data, overfitting may occur in specific scenarios or tasks, impacting its generalization ability across different environments and tasks.

Future research could further optimize image fusion methods through various approaches. Firstly, lighter network architectures could be explored to reduce reliance on computational resources, particularly for applications on edge devices. Secondly, to address the fusion challenges of low-quality images and high-noise environments, future work could consider introducing image pre-processing techniques. Overfitting could be mitigated through diverse training data or transfer learning methods. Finally, by integrating reinforcement learning and other technologies, the optimal fusion strategy could be selected more intelligently, enhancing the system’s adaptability and practical application value.

## Supporting information

S1 File Minimal data set definition. (DOCX)
