# Supplementary material for: A high-efficiency palmprint recognition model integrating ROI and Gabor filtering
Source: PLoS One. 2025 Jun 2;20(6):e0323373. doi: 10.1371/journal.pone.0323373 (PMC12129327; doi:10.1371/journal.pone.0323373)
Supplement: S1 File — (DOCX) [file pone.0323373.s001.docx]

**The data in Figure 7**

| CANNY-ROI | | SPEM-ROI | | RGB-ROI | |
| --- | --- | --- | --- | --- | --- |
| Dataset | SNR | Dataset | SNR | Dataset | SNR |
| 115 | 0.35 | 113 | 0.61 | 109 | 0.53 |
| 153 | 0.35 | 151 | 0.62 | 133 | 0.58 |
| 190 | 0.36 | 189 | 0.63 | 168 | 0.64 |
| 228 | 0.37 | 227 | 0.64 | 206 | 0.67 |
| 266 | 0.40 | 265 | 0.65 | 244 | 0.69 |
| 304 | 0.43 | 303 | 0.65 | 282 | 0.71 |
| 340 | 0.48 | 341 | 0.66 | 320 | 0.73 |
| 369 | 0.54 | 379 | 0.67 | 357 | 0.74 |
| 396 | 0.60 | 417 | 0.68 | 395 | 0.76 |
| 429 | 0.66 | 455 | 0.67 | 433 | 0.79 |
| 468 | 0.70 | 493 | 0.67 | 471 | 0.82 |
| 506 | 0.74 | 531 | 0.68 | 509 | 0.85 |
| 544 | 0.77 | 569 | 0.68 | 547 | 0.88 |
| 581 | 0.80 | 607 | 0.69 | 585 | 0.92 |
| 619 | 0.82 | 644 | 0.70 | 623 | 0.94 |
| 657 | 0.84 | 682 | 0.70 | 661 | 0.96 |
| 695 | 0.85 | 720 | 0.70 | 699 | 0.97 |
| 733 | 0.85 | 758 | 0.71 | 737 | 0.97 |
| 796 | 0.85 | 792 | 0.71 | 799 | 0.98 |
| Dataset | SIL | Dataset | SIL | Dataset | SIL |
| 122 | 0.27 | 122 | 0.36 | 122 | 0.31 |
| 169 | 0.26 | 169 | 0.35 | 169 | 0.30 |
| 216 | 0.25 | 216 | 0.34 | 216 | 0.28 |
| 263 | 0.24 | 263 | 0.31 | 259 | 0.25 |
| 310 | 0.23 | 306 | 0.28 | 301 | 0.21 |
| 355 | 0.22 | 344 | 0.24 | 348 | 0.20 |
| 404 | 0.21 | 376 | 0.21 | 395 | 0.19 |
| 451 | 0.18 | 401 | 0.17 | 442 | 0.19 |
| 498 | 0.15 | 424 | 0.13 | 489 | 0.18 |
| 545 | 0.13 | 458 | 0.10 | 536 | 0.17 |
| 592 | 0.11 | 502 | 0.07 | 583 | 0.16 |
| 639 | 0.10 | 549 | 0.06 | 630 | 0.16 |
| 685 | 0.09 | 596 | 0.05 | 677 | 0.15 |
| 732 | 0.08 | 737 | 0.04 | 723 | 0.14 |
| 778 | 0.07 | 780 | 0.04 | 770 | 0.14 |

**The data in Figure 8**

| CANNY-ROI | | SPEM-ROI | | RGB-ROI | |
| --- | --- | --- | --- | --- | --- |
| Iterations | SNR | Iterations | SNR | Iterations | SNR |
| 3 | 0.42 | 3 | 0.38 | 3 | 0.44 |
| 6 | 0.42 | 5 | 0.38 | 6 | 0.44 |
| 9 | 0.42 | 8 | 0.38 | 9 | 0.44 |
| 12 | 0.44 | 14 | 0.40 | 12 | 0.45 |
| 15 | 0.45 | 17 | 0.40 | 15 | 0.46 |
| 18 | 0.45 | 19 | 0.40 | 18 | 0.46 |
| 21 | 0.45 | 24 | 0.43 | 21 | 0.46 |
| 32 | 0.50 | 27 | 0.43 | 24 | 0.46 |
| 35 | 0.50 | 28 | 0.43 | 27 | 0.46 |
| 38 | 0.50 | 34 | 0.47 | 29 | 0.46 |
| 41 | 0.50 | 37 | 0.47 | 43 | 0.49 |
| 44 | 0.52 | 39 | 0.47 | 46 | 0.49 |
| 47 | 0.52 | 43 | 0.58 | 49 | 0.49 |
| 52 | 0.56 | 45 | 0.59 | 52 | 0.50 |
| 55 | 0.56 | 48 | 0.59 | 55 | 0.50 |
| 58 | 0.56 | 52 | 0.62 | 58 | 0.50 |
| 61 | 0.57 | 54 | 0.62 | 61 | 0.50 |
| 64 | 0.58 | 58 | 0.62 | 64 | 0.51 |
| 67 | 0.58 | 63 | 0.67 | 67 | 0.51 |
| 70 | 0.59 | 66 | 0.67 | 70 | 0.52 |
| 73 | 0.60 | 69 | 0.67 | 73 | 0.53 |
| 76 | 0.60 | 72 | 0.71 | 76 | 0.53 |
| 79 | 0.60 | 75 | 0.71 | 79 | 0.53 |
| 82 | 0.62 | 78 | 0.71 | 82 | 0.55 |
| 88 | 0.62 | 82 | 0.75 | 85 | 0.55 |
| 91 | 0.64 | 85 | 0.75 | 88 | 0.55 |
| 94 | 0.64 | 87 | 0.75 | 91 | 0.58 |
| 97 | 0.64 | 91 | 0.79 | 94 | 0.58 |
| 84 | 0.61 | 98 | 0.80 | 97 | 0.58 |
| CANNY-ROI | | SPEM-ROI | | RGB-ROI | |
| Iterations | RMSE | Iterations | RMSE | Iterations | RMSE |
| 3 | 0.32 | 2 | 0.30 | 3 | 0.33 |
| 7 | 0.32 | 4 | 0.31 | 7 | 0.33 |
| 10 | 0.32 | 7 | 0.31 | 11 | 0.33 |
| 12 | 0.28 | 12 | 0.27 | 15 | 0.33 |
| 16 | 0.28 | 15 | 0.27 | 19 | 0.33 |
| 20 | 0.27 | 18 | 0.27 | 23 | 0.32 |
| 24 | 0.27 | 25 | 0.26 | 27 | 0.32 |
| 28 | 0.27 | 28 | 0.26 | 31 | 0.31 |
| 32 | 0.25 | 31 | 0.24 | 35 | 0.31 |
| 36 | 0.25 | 42 | 0.23 | 39 | 0.31 |
| 40 | 0.25 | 46 | 0.23 | 43 | 0.31 |
| 44 | 0.24 | 48 | 0.23 | 47 | 0.31 |
| 48 | 0.24 | 52 | 0.22 | 51 | 0.30 |
| 52 | 0.23 | 55 | 0.22 | 55 | 0.30 |
| 56 | 0.23 | 61 | 0.20 | 59 | 0.30 |
| 60 | 0.23 | 64 | 0.20 | 63 | 0.30 |
| 64 | 0.22 | 68 | 0.20 | 67 | 0.30 |
| 68 | 0.22 | 72 | 0.17 | 71 | 0.29 |
| 72 | 0.21 | 75 | 0.18 | 75 | 0.29 |
| 76 | 0.21 | 86 | 0.15 | 79 | 0.29 |
| 80 | 0.21 | 89 | 0.15 | 83 | 0.29 |
| 84 | 0.20 | 91 | 0.13 | 87 | 0.29 |
| 88 | 0.20 | 94 | 0.13 | 92 | 0.28 |
| 92 | 0.19 | 98 | 0.13 | 96 | 0.28 |

**The data in Figure 9**

| Training set size | RGB-ROI | CANNY-ROI | SPEM-ROI |
| --- | --- | --- | --- |
| Training set 1 | 395 | 355 | 342 |
| Training set 2 | 384 | 344 | 331 |
| Training set 3 | 331 | 304 | 294 |
| Training set 4 | 322 | 265 | 252 |
|  |  |  |  |
| Verification set size | RGB-ROI | CANNY-ROI | SPEM-ROI |
| Validation set 1 | 100 | 165 | 158 |
| Validation set 2 | 141 | 200 | 221 |
| Validation set 3 | 165 | 267 | 304 |
| Validation set 4 | 200 | 321 | 302 |

**The data in Figure 10**

| RF-Gabor | | Gabor-SVM | | KNN-Gabor | |
| --- | --- | --- | --- | --- | --- |
| Iterations | ACC | Iterations | ACC | Iterations | ACC |
| 5 | 14.03 | 5 | 10.80 | 4 | 10.28 |
| 6 | 19.73 | 6 | 13.94 | 5 | 12.94 |
| 7 | 25.24 | 7 | 17.28 | 6 | 15.59 |
| 8 | 30.55 | 8 | 20.46 | 7 | 17.98 |
| 9 | 35.87 | 9 | 23.86 | 8 | 21.02 |
| 10 | 41.38 | 10 | 26.81 | 9 | 22.89 |
| 11 | 46.86 | 11 | 30.28 | 10 | 24.87 |
| 12 | 52.86 | 12 | 33.44 | 11 | 26.85 |
| 13 | 59.54 | 13 | 36.99 | 12 | 28.42 |
| 14 | 65.04 | 14 | 40.53 | 13 | 29.92 |
| 15 | 69.74 | 15 | 43.90 | 14 | 31.31 |
| 16 | 73.46 | 16 | 47.73 | 15 | 32.78 |
| 17 | 76.02 | 17 | 51.10 | 16 | 34.62 |
| 18 | 77.99 | 18 | 55.06 | 17 | 36.39 |
| 19 | 79.49 | 19 | 59.53 | 18 | 38.46 |
| 20 | 80.64 | 20 | 64.06 | 19 | 40.43 |
| 21 | 81.33 | 21 | 68.68 | 20 | 42.53 |
| 22 | 81.91 | 22 | 72.64 | 21 | 44.68 |
| 23 | 82.31 | 23 | 77.18 | 22 | 47.28 |
| 24 | 82.49 | 24 | 80.94 | 23 | 50.02 |
| 25 | 82.59 | 25 | 83.55 | 24 | 52.63 |
| 26 | 82.68 | 26 | 85.85 | 25 | 55.75 |
| 27 | 82.68 | 27 | 87.68 | 26 | 58.95 |
| 28 | 82.68 | 28 | 89.09 | 27 | 62.28 |
| 29 | 82.68 | 29 | 90.61 | 28 | 65.50 |
| 30 | 82.68 | 30 | 91.69 | 29 | 68.08 |
| 31 | 82.68 | 31 | 92.42 | 30 | 70.80 |
| 32 | 82.68 | 32 | 93.44 | 31 | 73.07 |
| 33 | 82.78 | 33 | 94.08 | 32 | 74.85 |
| 34 | 82.78 | 34 | 94.45 | 33 | 76.28 |
| 35 | 82.88 | 35 | 95.04 | 34 | 77.67 |
| 36 | 82.88 | 36 | 95.44 | 35 | 79.08 |
| 37 | 82.88 | 37 | 95.77 | 36 | 79.76 |
| 38 | 82.88 | 38 | 96.04 | 37 | 80.43 |
| 39 | 82.97 | 39 | 96.21 | 38 | 81.12 |
| 40 | 82.97 | 40 | 96.32 | 39 | 81.73 |
| 41 | 82.97 | 41 | 96.41 | 40 | 82.10 |
| 42 | 82.97 | 42 | 96.50 | 41 | 82.60 |
| 43 | 82.95 | 43 | 96.51 | 42 | 83.35 |
| 44 | 82.88 | 44 | 96.51 | 43 | 83.89 |
| 45 | 82.80 | 45 | 96.51 | 44 | 84.43 |
| 46 | 82.68 | 46 | 96.41 | 45 | 84.99 |
| 47 | 82.59 | 47 | 96.42 | 46 | 85.52 |
| RF-Gabor | | Gabor-SVM | | KNN-Gabor | |
| Iterations | ACC | Iterations | ACC | Iterations | ACC |
| 2 | 0.88 | 2 | 0.87 | 3 | 0.87 |
| 3 | 0.85 | 3 | 0.69 | 4 | 0.86 |
| 4 | 0.80 | 4 | 0.60 | 5 | 0.84 |
| 5 | 0.76 | 5 | 0.49 | 6 | 0.82 |
| 6 | 0.74 | 6 | 0.42 | 7 | 0.81 |
| 7 | 0.68 | 7 | 0.34 | 8 | 0.79 |
| 8 | 0.65 | 8 | 0.31 | 9 | 0.77 |
| 9 | 0.62 | 9 | 0.27 | 10 | 0.76 |
| 10 | 0.58 | 11 | 0.24 | 11 | 0.74 |
| 11 | 0.55 | 12 | 0.21 | 12 | 0.71 |
| 12 | 0.57 | 13 | 0.19 | 13 | 0.68 |
| 13 | 0.49 | 15 | 0.17 | 14 | 0.66 |
| 14 | 0.53 | 16 | 0.16 | 15 | 0.61 |
| 15 | 0.43 | 17 | 0.14 | 16 | 0.55 |
| 16 | 0.42 | 19 | 0.13 | 17 | 0.46 |
| 17 | 0.39 | 20 | 0.12 | 18 | 0.35 |
| 20 | 0.30 | 21 | 0.11 | 19 | 0.29 |
| 21 | 0.29 | 23 | 0.10 | 20 | 0.26 |
| 22 | 0.29 | 24 | 0.10 | 21 | 0.25 |
| 23 | 0.26 | 25 | 0.09 | 22 | 0.23 |
| 24 | 0.27 | 27 | 0.08 | 23 | 0.22 |
| 28 | 0.25 | 28 | 0.08 | 24 | 0.22 |
| 29 | 0.25 | 29 | 0.08 | 27 | 0.21 |
| 30 | 0.26 | 31 | 0.07 | 28 | 0.21 |
| 31 | 0.26 | 32 | 0.07 | 29 | 0.20 |
| 32 | 0.25 | 33 | 0.07 | 30 | 0.20 |
| 33 | 0.25 | 35 | 0.07 | 31 | 0.19 |
| 34 | 0.25 | 36 | 0.07 | 32 | 0.19 |
| 35 | 0.25 | 37 | 0.07 | 35 | 0.18 |
| 36 | 0.25 | 39 | 0.07 | 36 | 0.18 |
| 37 | 0.25 | 40 | 0.06 | 37 | 0.17 |
| 38 | 0.25 | 41 | 0.07 | 38 | 0.17 |
| 39 | 0.25 | 43 | 0.07 | 39 | 0.17 |
| 40 | 0.25 | 44 | 0.07 | 40 | 0.17 |
| 41 | 0.25 | 45 | 0.07 | 41 | 0.16 |
| 42 | 0.25 | 47 | 0.07 | 43 | 0.16 |
| 43 | 0.25 | 48 | 0.07 | 44 | 0.15 |
| 44 | 0.25 | 49 | 0.07 | 45 | 0.15 |

**The data in Figure 11**

| GPDS | | | |
| --- | --- | --- | --- |
| Different types | Gabor-SVM | KNN-Gabor | RF-Gabor |
| Group 1 | 0.48 | 0.58 | 0.55 |
| Group 2 | 0.24 | 0.32 | 0.41 |
| Group 3 | 0.33 | 0.37 | 0.44 |
| Group 4 | 0.28 | 0.30 | 0.32 |
| Group 5 | 0.18 | 0.19 | 0.41 |
| CASIA | | | |
| Different types | Gabor-SVM | KNN-Gabor | RF-Gabor |
| Group 1 | 0.15 | 0.31 | 0.18 |
| Group 2 | 0.59 | 0.82 | 0.67 |
| Group 3 | 0.14 | 0.37 | 0.22 |
| Group 4 | 0.38 | 0.41 | 0.61 |
| Group 5 | 0.34 | 0.38 | 0.41 |
